# Mid-term results of a physiotherapist-led Ponseti service for the management of non-idiopathic and idiopathic clubfoot

**DOI:** 10.1007/s11832-015-0658-8

**Published:** 2015-06-14

**Authors:** Mia Dunkley, Yael Gelfer, Debbie Jackson, Evette Parnell, Jennifer Armstong, Cristina Rafter, Deborah M. Eastwood

**Affiliations:** Department of Physiotherapy, Great Ormond St Hospital for Children, London, WC1N 3JL UK; Department of Orthopaedics, Great Ormond St Hospital for Children, London, WC1N 3JL UK; Department of Orthopaedics, St George’s Hospital, London, SW17 0QT UK

**Keywords:** Non-idiopathic, Clubfoot, Ponseti, Physiotherapist

## Abstract

**Background:**

The Ponseti method is the preferred treatment for idiopathic clubfoot. Although popularised by orthopaedic surgeons it has expanded to physiotherapists and other health practitioners. This study reviews the results of a physiotherapist-led Ponseti service for idiopathic and non-idiopathic clubfeet and compares these results with those reported by other groups.

**Method:**

A prospective cohort of clubfeet (2005–2012) with a minimum 2-year follow-up after correction was reviewed. Physiotherapists treated 91 children—41 patients (69 feet) had non-idiopathic deformities and 50 children (77 feet) were idiopathic. Objective outcomes were evaluated and compared to results from other groups managing similar patient cohorts.

**Results:**

The mean follow-up was 4.6 years (range 2–8.3 years) for both groups. The non-idiopathic group required a median of 7 casts to correct the clubfoot deformity with an 83 % tenotomy rate compared to a median of 5 casts for the idiopathic group with a 63 % tenotomy rate. Initial correction was achieved in 96 % of non-idiopathic feet and in 100 % of idiopathic feet. Recurrence requiring additional treatment was higher in the non-idiopathic group with 40 % of patients (36 % of feet) sustaining a relapse as opposed to 8 % (6 % feet) in the idiopathic group. Surgery was required in 26 % of relapsed non-idiopathic feet and 6 % of idiopathic.

**Conclusions:**

Although Ponseti treatment was not as successful in non-idiopathic feet as in idiopathic feet, deformity correction was achieved and maintained in the mid-term for the majority of feet. These results compare favourably to other specialist orthopaedic-based services for Ponseti management of non-idiopathic clubfeet.

**Level of evidence:**

Prognostic Level III.

## Introduction

Historically, treatment of idiopathic clubfoot deformity has used both non-operative methods of manipulation and splinting [1, 2] and extensive soft-tissue releases [3–5]. Each method can achieve initial correction of the deformity but surgical procedures give variable results in the short-term [5], moderate results in the longer term (with less invasive surgical procedures) [3], and poor long-term results in the majority of feet following extensive soft-tissue releases [6].

The Ponseti method of clubfoot management for the idiopathic foot has a high success rate [7–12] with long-term follow-up results superior to those following surgery [13] and, consequently, the Ponseti method is now the treatment of choice. Physiotherapists have replicated this success [8, 10] and, furthermore, have been shown to deliver this service with fewer recurrences when compared to orthopaedic surgeons [9].

However, until recently, clubfeet associated with neuromuscular conditions or other syndromes, known as non-idiopathic clubfeet, have been considered resistant to non-operative management [14–17]. A few recent studies evaluated the results of the Ponseti method in orthopaedic-based services treating non-idiopathic clubfeet [14–18]; however, there are no reports in the literature from a physiotherapist-based Ponseti clinic.

At our institution, a physiotherapist-led team manages all referrals for both non-idiopathic and idiopathic clubfeet. The aim of this prospective study was to ascertain whether the mid-term results of a physiotherapist-led Ponseti service could equal those reported by medical-led services for both non-idiopathic and idiopathic clubfeet.

## Methods

This study was conducted at a tertiary referral centre for paediatric services. In 2005, a Ponseti clinic was developed and established by a physiotherapist in conjunction with the support of the orthopaedic surgeon and the institution. The lead physiotherapist is a clinical specialist with 15 years of training in paediatric orthopaedics at both primary and tertiary referral centres. Her Ponseti training and her general experience with paediatric orthopaedic conditions in combination with her understanding of musculoskeletal development enable her to fulfil this role.

All clubfoot referrals were directed to the Ponseti service and, if medically stable, all patients were seen within 2 weeks. All appointments were led by the physiotherapist and the orthopaedic surgeon did not review idiopathic feet unless surgery was indicated or medical complications had arisen. All non-idiopathic clubfeet were discussed with the orthopaedic surgeon and an individual management plan devised. When necessary, an orthopaedic review was arranged in consultant-led clinics that ran concurrently with the Ponseti clinic.

At the initial consultation, the physiotherapist performed a full medical assessment including a detailed foot examination. Standardised photographs were taken. Each patient signed a consent form for measurement collection, photographic documentation and the use of data for presentations. IRB approval had been obtained.

All feet were scored at each attendance with the Pirani score. The ankle and subtalar ranges of movement were noted and each patient’s evertor muscle function was documented using a scoring system modelled on the Pirani score in accordance with departmental policy [8, 19, 20] (Table 1).

**Table 1:** Evertor activity

Muscle activity grade	Movement observed^a^
0	Sustained/normal toe dorsiflexion and foot eversion
0.5	Some foot evertor activity and/or toe flaring
1	No toe flaring or foot eversion

^a^Toe dorsiflexion and foot evertor movement noted in response to stroking the sole or the dorsolateral aspect of the calf/foot

**Fig. 1:**
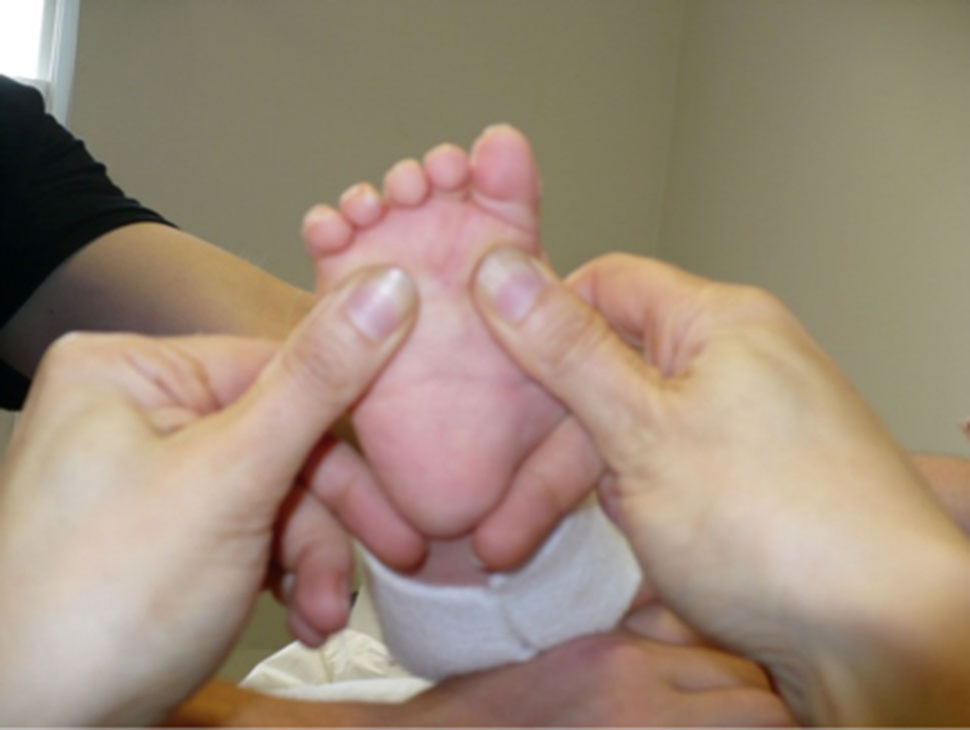
Position of hands to correct the hyperflexion and equinus simultaneously in the complex feet

Treatment options were explained and with family agreement, manipulation and application of a well-moulded above-knee cast was performed by the physiotherapist and her assistant at weekly intervals, following the Ponseti protocol [21, 22]. Feet defined as complex on referral were treated according to the Ponseti approach for complex feet [15]. Precise identification of the subtalar joint was important in complex idiopathic and non-idiopathic feet, so that movement of this joint could be felt when the foot was slowly abducted during correction. In order to avoid slippage of the plaster casts in complex feet, hyperflexion of the metatarsals and the rigid equinus were addressed simultaneously (Fig. 1) [15].

**Fig. 2:**
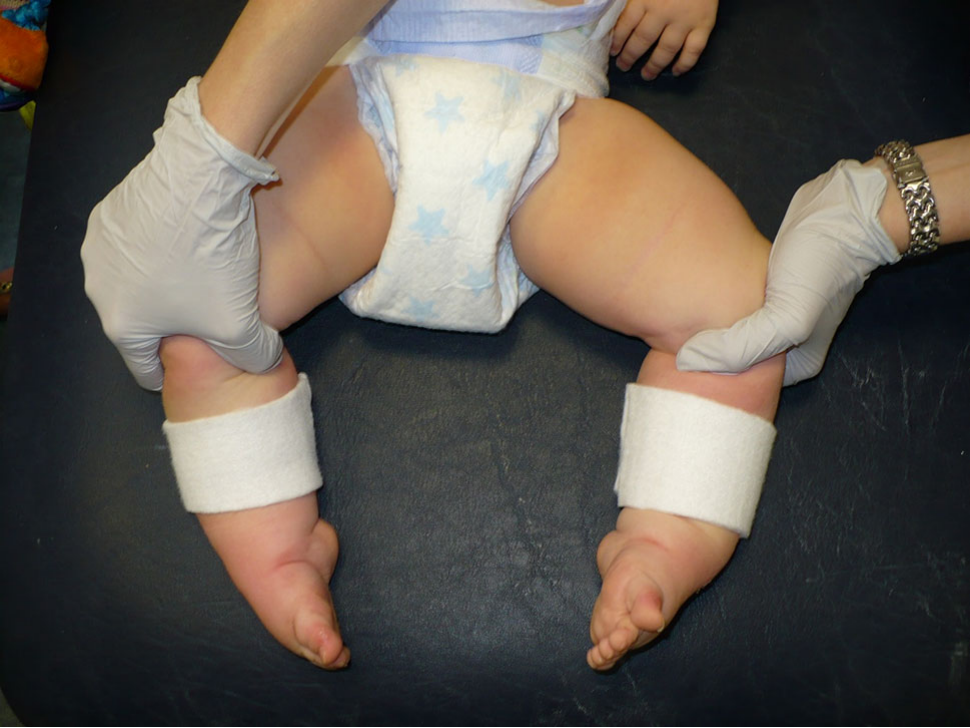
Position of felt applied to calf to help prevent cast slippage in cases of severe equinus and/or limited knee flexion

When the management plan included simultaneous treatment of congenital knee deformities, the protocol was adjusted. If there was minimal knee flexion and/or marked equinus, an additional strip of adhesive felt padding was applied directly to the patient’s skin to help secure the plaster to minimise slippage (Fig. 2). If the skin became red under the adhesive pad, the site of the pad was changed to rest the skin; all erythematous areas resolved by the subsequent cast change. All patients were comfortable in their casts. If there was a co-existing developmental dysplasia of the hip then priority was given to the hip and the feet were plastered simultaneously if possible.

A percutaneous tenotomy of the Achilles tendon (TAT) was performed by a surgeon to address residual equinus (<15° of dorsiflexion with the knee extended) under local anaesthetic in the outpatient clinic in babies aged <8 months, or in the operating room in older infants. The TAT was arranged by the physiotherapist but the need for tenotomy was confirmed by the surgeon immediately prior to the procedure as part of the consent process. Following tenotomy, the foot was cast for 3 weeks in the fully corrected position. If necessary, complex feet were recast during the 3-week period to achieve full dorsiflexion. The number of casts required and the tenotomy rates were noted. Failure of the Ponseti method was defined as an inability to achieve full correction of the clubfoot with initial casting with or without a tenotomy.

Parents were instructed to wear the boots and bars for 23 h a day for 3 months followed by 12–14 h at night and nap times, reducing to night time only between years 3–5. Patients in the non-idiopathic group were also supplied with other appropriate orthoses and walking aids in keeping with their developmental status.

All patients were reviewed after the first week of using the boots and bars to ascertain compliance and identify potential problems. Thereafter, they were reviewed at 6 weeks and then at 3-monthly intervals until 5 years of age. Compliance was measured at each consultation by the parent report, observation of splint ‘wear’ and the ease with which the parents could apply the splint in the clinic. Any suggestion of poor compliance increased the frequency of clinic visits and the importance of compliance in relation to outcome was stressed. All families were given an information sheet, which included contact numbers and open access to the Ponseti clinic in cases of parental concern.

At each follow-up, the patients were assessed for signs of relapse defined as a deterioration in any component of the deformity (cavus, adductus, varus or equinus) that required further treatment; therefore, a lack of ankle dorsiflexion with the knee extended or subtalar joint range of motion of <30° was considered a relapse (i.e., loss of eversion). An orthopaedic follow-up was arranged by the physiotherapist for feet that relapsed and that might require surgical intervention. The orthopaedic surgeon decided what, if any, surgery was required.

Non-attenders were contacted by a team member and repeated non-attendance discussed with the medical team(s).

Orthopaedic review for the non-idiopathic feet continued at defined time points to discuss overall patient management.

## Results

Between September 2005 and December 2012, 105 children less than walking age (12 months) were treated in the Ponseti clinic. Ten patients were lost-to-follow-up and 4 (in the non-idiopathic group) died; the remaining 91 patients (146 feet) completed a minimum 2-year follow-up. Eight of 91 patients had received some casting treatment prior to referral; however, none had undergone TAT.

The foot deformities were classified as non-idiopathic in 41 patients (69 feet) and as idiopathic in the remaining 50 patients (77 feet). In the non-idiopathic group, 58 % were male versus 66 % in the idiopathic group. Sixty-seven percent of cases in the non-idiopathic group were bilateral compared to 58 % in the idiopathic group. The mean age at presentation for both groups was 2.8 months (range 3 days–11 months) and the mean duration of follow-up from deformity correction and fitting of boots and bars was 4.6 years (range 2–8.3 years) (Table 2).

**Table 2:** Demographic details of both patient cohorts

	Non-idiopathic (69 feet in 41 patients)	Idiopathic (77 feet in 50 patients)	Significance
Male:female	24:17	33:17	NS
Percentage bilateral	67 %	58 %	NS
Mean age (range) at onset of treatment	2.8 months (3 days to 9 months)	2.8 months (10 days to 11 months)	NS
Mean duration (range) of follow-up (post correction)	4.6 years (2–8 years)	4.9 years (2–8.3 years)	NS
Mean Pirani score (range) at treatment onset	5.5 (3–6)	5.0 (2.5–6)	NS
Compliance (%)	80 %	80 %	NS
Median number of casts (range)	7 (1–19)	5 (1–13)	NS
Feet with a poor Evertor score (0.5 or 1)	55/67 (2 not recorded)	26/77	*p* = 0.0001
Tenotomy (%)	83 %	63 %	*p* = 0.009

The aetiology of the deformity in non-idiopathic cases is given in Table 3. The most common aetiology was spinal dysraphism (15 cases).

**Table 3:** Aetiology of foot deformity in the non-idiopathic group

Aetiological grouping	No of patients (feet)	No of Ponseti failures patients (feet)	Recurrent deformity
Spinal dysraphism	15 (24)	1 (2)	13/22 feet (60 %)
Skeletal dysplasia	9 (15)		3/15 feet (20 %)
Arthrogryposis	7 (13)	1 (1)	2/12 feet (17 %)
Other syndromes	5 (8)		None
Other neurological problems	4 (7)		4/7
Complex amniotic band	1 (2)		2/2

### Number of casts and tenotomy rates (Table 2)

The non-idiopathic group had a median Pirani score of 5.5 (compared to 5 in the idiopathic group) and required a mean of 7 casts compared to a mean of 5 casts for the idiopathic group to correct the clubfoot deformity. The tenotomy rate varied between groups—83 % non-idiopathic versus 63 % idiopathic. One non-idiopathic foot in one patient required an early repeat TAT (initial tenotomy incomplete). Twenty-five complex feet (9 idiopathic and 16 non-idiopathic) required additional casting (in the immediate post-tenotomy period) to achieve acceptable dorsiflexion.

### Failure of Ponseti management

Initial correction was achieved in 96 % of the feet in the non-idiopathic group and in 100 % of feet in the idiopathic group. The compliance rate with the boots and bars treatment phase was considered either complete or poor. In the clinic, non-compliance was suspected from the parent report, boot appearance and the ability of the parents to fit the feet into the boots easily whilst being observed. In both groups, 20 % of families had difficulties with compliance particularly after the age of 4 years. In the idiopathic group, the majority of ‘non-compliance’ cases related to night time sleeping patterns that were <10 h.

The 3 feet (2 patients) with initial failure of correction in the non-idiopathic group were treated surgically. One patient with arthrogryposis multiplex congenital underwent a peritalar release at 12 months of age (Ponseti treatment had been successful on the contralateral foot). The second patient with spinal dysraphism underwent bilateral talectomies with soft-tissue releases when aged 2 years 3 months.

### Recurrent deformity

Recurrent deformity requiring treatment was high in the non-idiopathic group. Thirty-six percent of corrected non-idiopathic feet relapsed (24 feet in 16 patients) as opposed to 6 % (5 feet in 4 patients) in the idiopathic group (*p* < 0.0001). Treatment varied with the type and extent of the recurrence, the age at which it presented and the overall medical condition of the child.

In the non-idiopathic group, 24 corrected feet in 16 patients relapsed at a mean of 3.2 years (range 4.5 months to 7.6 years) after initial correction. The treatments given are shown in Table 4. Overall, in this group, 82 % of feet showed a reduction in evertor muscle function (grade 0.5/1) [8] at the time of deformity correction and application of boots and bars. Twenty-two of the 24 recurrences occurred in patients with poor evertor muscle function. Overall, of the 48 patients with poor evertor muscle function at the completion of treatment, 22 (46 %) relapsed. Recurrence was most likely in the group with spina bifida.

**Table 4:** Non-idiopathic group: treatment of first relapse

Treatment	Number of feet	Outcome
Recast only	13	9/13 feet second recurrence
Recast and TAT	2	2/2 feet second recurrence
Recast and TATT	5	Correction maintained
Recast and TATT and TAT	2	Correction maintained
Holistic management—no treatment offered	2	

*TATT* tibialis anterior tendon transfer, *TAT* tenotomy of the Achilles tendon

Eleven feet in 7 patients had a second relapse at a mean of 2.7 years following successful treatment of the first relapse. Nine of these 11 feet that relapsed for a second time had undergone recasting only at the first relapse (Table 4). The treatment of the second relapse is shown in Table 5.

**Table 5:** Non-idiopathic group: treatment of the second relapse

Treatment	Number of feet	Outcome
Recast only	4	4/4 feet third recurrence
Recast, TATT and TAT	1	
Peritalar release	4	
Talectomy	1	
Holistic management—no treatment offered	1	

*TATT* tibialis anterior tendon transfer, *TAT* tenotomy of the Achilles tendon

Four feet in 2 patients had a third relapse at a mean of 1.3 years following successful treatment of the second relapse; all 4 feet underwent a peritalar release and deformity correction.

In 3 patients (3 feet), as part of a holistic approach to patient care and their general medical condition, the recurrent deformity was not treated.

In the idiopathic group, 5 feet in 4 patients relapsed at a mean of 4.6 years (range 2.3–6.8 years) after initial correction. One foot was recast and the other 4 feet underwent tibialis anterior tendon transfer (TATT). The foot that was only recast suffered a second relapse 10 months later and underwent TATT. Overall, 33 % of feet in this idiopathic group showed a reduction in evertor muscle function (grade 0.5) [8]. All 5 relapsed feet had poor evertor function and 3 of the 4 patients had shown poor compliance with the boots and bars programme.

### Complications

A total of 7 patients (4 non idiopathic feet and 3 idiopathic feet) had pressure-related problems in their boots and bars, 5 with EPUAP (European Pressure Ulcer Advisory Panel) stage 1 and 2 with stage 3 sores at the back of their heels. Both stage 3 sores resolved with dressings and time out of boots and bars. The majority of non-idiopathic feet were managed in the soft Mitchell boots and bars (C-Pro Direct Ltd, Kent, UK) as the feet were held securely and the position of the heel could be checked easily. Semeda boots and bars (Semeda E.K., Stoetze, Germany) with soft heel inserts were used if pressure problems were experienced. Idiopathic feet were generally held in the Markell boots (Kettering Surgical Appliances Ltd, Northampton, UK) as they were more cost-effective and similar clinical results were seen with this patient group [23].

## Discussion

Using the Ponseti method, both orthopaedic surgeons and physiotherapists have demonstrated excellent outcomes for children with idiopathic clubfoot [8–12]. Our initial correction rate of 100 % for idiopathic clubfeet compares favourably with other series that report success rates of 83–98 % [8, 10, 11, 21, 24]. Six percent of feet in the idiopathic cohort relapsed and all eventually required TATT; this compares well with the 11 % relapse rate reported by Morcuende et al. [25], 13 % by Janicki et al. [16] and 8 % by Moroney et al. [17]. These 3 series [16, 17, 25] all report a 2–6 % requirement for extensive surgical release; however, none of our idiopathic feet required such surgery.

Until recently, non-idiopathic clubfeet have been considered resistant to non-operative management [14–17]. These feet (and indeed the patient) present additional treatment challenges and results of treatment are inferior to those for idiopathic feet with higher rates for both recurrence and surgery [16–18, 26]. Although our results agree with this, correction of the initial deformity was achieved and maintained in the mid-term in 61 % of feet (56 % patients). There are only a few studies reporting the early results of the Ponseti method in orthopaedic-based services treating non-idiopathic clubfeet [14–18]; however, some of these were disease–specific studies and may not be representative of all non-idiopathic TEV foot pathology. Only 2 studies were found in the literature for direct comparison [16, 17]. These showed initial success rates of 90 and 91 %, which compare to our success rate of 96 %. In both series, 44 % of feet relapsed which is marginally higher than our 36 % relapse rate and their surgical rates of 28 and 37 % are in keeping with our rate of 26 %. Both these studies [16, 17] had a minimum 1-year follow-up period and acknowledged that further relapses were likely especially as the majority of relapses occur in the first 2 years; our minimum follow-up period is 2 years. Further relapses are anticipated through the growing years of these children with complex problems but management will be directed by the individual child’s prognosis for walking and standing.

The higher recurrence rate in non-idiopathic feet is thought to be attributed to a more severe initial deformity, lack of initial full correction compounded by the absence of weight bearing, as described by Gerlach et al. [14]. Additionally, weak muscle power around the ankle is thought to be a contributing factor [19, 27].

As anticipated, non-idiopathic clubfeet required more casts (median 7 versus 5) to achieve initial correction. Overall, the median numbers of casts required has reduced, with experience, in the idiopathic cohort but not in the non-idiopathic group [19]. Our TAT rate is also higher in the non-idiopathic series (83 versus 63 %). Our low tenotomy rate in the idiopathic group is in keeping with other reported physiotherapist-delivered services for idiopathic feet where the rates were 62.5 % [8] and 46 % [10]. In our idiopathic feet with an initial Pirani score >4.0, the tenotomy rate was 86 %; there have been no relapses in idiopathic feet that have not undergone a tenotomy.

In an earlier series with a shorter mean follow-up we reported higher compliance rates with the boots and bars regime [19]; however, this has fallen as the mean age of the patient has increased. In non-idiopathic feet, some patients have changed to ankle foot orthoses as part of their overall care plan. In idiopathic feet, it has been difficult to keep the older child compliant for 10 h per night. Three of 4 children with recurrent idiopathic deformities were non-compliant but all five feet had poor evertor muscle function. Previous studies have documented that a failure to follow the boots and bars regime is associated with relapse [28, 29], and whilst we know that boots and bars have a major influence on outcome, we do not believe that non-compliance is the sole aetiological cause for the recurrent deformity [19]. We suggest, like Zhao et al. [30], that brace protocols could be individualised to the patient, particularly for milder idiopathic feet in the older toddler.

In relapsed idiopathic feet, 1–3 stretching casts to regain subtalar joint eversion preceded all TATT procedures but no foot required a posterior capsular release. The one relapsed foot treated by casting alone, relapsed again and we now consider that all children who relapse and who are old enough to undergo TATT should do so at the first recurrence especially in the presence of weak evertor muscle function.

In the non-idiopathic group, it is important to recognise that the aims of treatment may be different and emphasis on the overall approach to the child’s well-being may determine whether or not a recurrence is treated and, if so, whether this is performed surgically or by repeated casting.

It has been reported that a dedicated Ponseti clinic improves outcomes [31] and that physiotherapy-led clubfoot clinics have been successful as physiotherapists possess the knowledge and skills necessary to implement the Ponseti method [8–10]. The optimal or minimal training period for a physiotherapist to acquire efficiency is unknown. At our hospital only specialist paediatric orthopaedic physiotherapists manage these patients; all have a minimum 10 years experience in paediatric orthopaedics ensuring high standards of orthopaedic knowledge and care. Continuity of care has been shown to correlate to increasing trust [8] and all families are well known to the practitioner at the time of application of the orthosis and throughout their care.

Our mid-term results with a physiotherapist-led Ponseti service for the management of non-idiopathic clubfoot are as good as those from other published series from orthopaedic-led services. When compared with idiopathic clubfeet, non-idiopathic clubfeet required more casts and had a higher rate of failures, recurrences, and additional procedures than idiopathic clubfeet but major surgical procedures were avoided in 70 % of cases.
